# Integrated Care for Latent Tuberculosis Infection (LTBI) at a Primary Health Care Facility for Refugees in Winnipeg, Canada: A Mixed-Methods Evaluation

**DOI:** 10.3389/fpubh.2019.00057

**Published:** 2019-03-21

**Authors:** Dione Benjumea-Bedoya, Marissa Becker, Margaret Haworth-Brockman, Shivoan Balakumar, Kimberly Hiebert, Jo-Anne Lutz, Alison Bertram Farough, Yoav Keynan, Pierre Plourde

**Affiliations:** ^1^Facultad Nacional de Salud Pública, University of Antioquia, Medellín, Colombia; ^2^National Collaborating Centre for Infectious Diseases, Rady Faculty of Health Sciences, University of Manitoba, Winnipeg, MB, Canada; ^3^BridgeCare Clinic, Winnipeg Regional Health Authority, Winnipeg, MB, Canada; ^4^Integrated Tuberculosis Services, Winnipeg Regional Health Authority, Winnipeg, MB, Canada; ^5^Department of Community Health Sciences, Rady Faculty of Health Sciences, University of Manitoba, Winnipeg, MB, Canada; ^6^Department of Medical Microbiology and Infectious Diseases, Rady Faculty of Health Sciences, University of Manitoba, Winnipeg, MB, Canada

**Keywords:** refugee health, latent TB, treatment completion, integrated management, LTBI evaluation

## Abstract

Although Canada has one of the lowest tuberculosis incidence rates in the world, certain groups are disproportionately affected, including foreign born people from high incidence countries. The Winnipeg Regional Health Authority has initiated a process to decentralize latent tuberculosis infection (LTBI) management at primary care clinics in Winnipeg. One of these clinics is BridgeCare Clinic which provides services to government-assisted refugees. The present study describes the BridgeCare Clinic LTBI program and reviews program outcomes from January 2015 to October 2016. Refugees at BridgeCare Clinic receive comprehensive care, including LTBI screening and treatment. The LTBI program is managed by physicians, nurse practitioners, and primary care nurses under a patient-centered model of care. An accessible interpretation service, education to clients, and laboratory sampling at the clinic with free IGRA testing are important components of the program. Anonymized data on client outcomes were statistically analyzed and qualitative interviews were conducted with senior staff. During the study period, 274 IGRA tests were ordered with 158 negative results (57.7%) and 101 positive results (36.9%). Of 45 clients eligible (from January to December 2015) for LTBI treatment, 11 (24.4%) declined to receive treatment and 34 (75.6%) started treatment. Twenty-seven (79.4%) clients completed treatment, 3 (8.8%) clients moved out of province, and 4 (11.8%) did not complete treatment. The most recent World Health Organization strategy for tuberculosis control calls for integrated, patient-centered care and prevention. Aligned with these WHO recommendations, our experience suggests that LTBI care and treatment can be delivered effectively in a primary care setting using an integrated patient-centered approach.

## Introduction

Although Canada has one of the lowest tuberculosis (TB) incidence rates in the world, certain groups are disproportionately affected, including foreign born people from high TB incidence countries (1). In 2016, TB among the foreign-born population accounted for 70% of reported cases (incidence rate of 15.2 per 100,000 population) in Canada, despite foreign-born residents representing only 22% of the total population (1). Persons from high prevalence regions of Africa and South East Asia had the highest rates, 45.1 and 34.9 per 100,000 population, respectively. The two countries of origin that accounted for the most cases were India (n = 257, 21.2% of all foreign-born cases), and the Philippines (n = 252, 20.8% of all foreign-born cases) (1).

Contrary to national figures, the foreign-born population accounted for only 33.8% of cases in the province of Manitoba, as Indigenous persons accounted for 61.7% of the cases (incidence rates were 30.8 and 53.9 per 100,000 population, respectively). Among foreign-born cases, the most frequent place of origin was the Western Pacific Region, with 38 of 68 cases (55.9%) (1).

A key strategy to control TB is screening and treatment for latent tuberculosis infection (LTBI). In accordance with the *Canadian Tuberculosis Standards*, LTBI treatment is self-administered isoniazid (INH), taken daily for 9 months (9INH) (2). Most LTBI programs strive to achieve treatment acceptance and completion rates of at least 80% (2). However, there are challenges to achieving these goals, including but not limited to, linguistic, cultural, socio-economic and health services barriers to providing adequate TB care (3), and to helping patients complete treatment. The World Health Organization (WHO) promotes TB care that is integrated with primary care (4). Various models of integrated primary and specialist care have been tried to optimize LTBI management, however no single approach has been found to be most effective in achieving better outcomes (5).

The Winnipeg Regional Health Authority (WRHA) (Winnipeg is the capital and largest city in the province of Manitoba) is moving toward integrated care, gradually decentralizing certain TB services since 2006. Complex LTBI cases continue to be assessed by a specialist at the Health Sciences Center teaching hospital. For clients without complicating medical conditions or drug interactions contraindicating INH, LTBI treatment is distributed among select primary care clinics in Winnipeg.

BridgeCare Clinic, a primary healthcare facility for new refugees, is one of these clinics (6). BridgeCare Clinic opened in 2010 to provide comprehensive primary care for government-assisted refugees during their first year in Winnipeg. It serves ~ 500 refugee clients every year. Clients can be assisted by a trained interpreter, and an outreach worker is available to provide information regarding health systems in Canada. A provincial population-based analysis on LTBI treatment completion outcomes in Manitoba (2012 to 2014) indicated that Winnipeg's LTBI primary care sites (including BridgeCare Clinic), were at least as successful as specialist clinics in achieving good treatment outcomes (>75% completion rates) (7). The analysis was based on aggregate data from Manitoba's Drug Program Information Network (8) across all sites. To follow up on these promising findings and to understand contributing factors better, we conducted an evaluation of patient uptake and outcomes of integrated LTBI screening and treatment at BridgeCare Clinic from January 2015 to October 2016.

## Methods

A mixed-methods approach was used for this program evaluation (9).

De-identified data from January 2015 to October 2016 were analyzed, including basic demographic information, numbers of clients screened with QuantiFERON®-TB Gold Interferon Gamma Release Assay (IGRA), and numbers receiving LTBI treatment. Global IGRA test result proportions were calculated, and bivariate analyses were conducted by sex, country of birth, and mean age. Country of birth was categorized to one of six WHO regions (10). Student's *t*-tests were used to compare quantitative variables, and Chi-Squared tests were used to compare qualitative variables, using 95% confidence.

Only IGRA test positive results from 2015 were included in the analysis of treatment completion (in order to allow for assessment of treatment completion), which was defined as receiving greater than or equal to 80% of INH doses within a 12-month period.

LTBI care cascades can help identify steps where losses of diagnosis and treatment occur for a particular population (11). To further support the analysis of LTBI care and treatment outcomes at BridgeCare Clinic, an LTBI care cascade for refugees in Manitoba was characterized. The refugee newcomer population to Manitoba during 2015 was used as the reference population, followed by government assisted refugees in Manitoba, and BridgeCare Clinic LTBI program data during the same year.

Descriptive data were gathered from document reviews and five semi-structured interviews with BridgeCare Clinic and WRHA staff. Using a standard script of questions, participants were asked their professional opinions about factors that facilitated or prevented patients completing treatment. Interviews were conducted in person, and participants' comments were recorded by the principal researcher by hand. Participants were able to review the notes before their inclusion in this analysis.

The facilitators and barriers for LTBI care identified at BridgeCare Clinic through interviews were categorized according to the five levels of the social ecological model (SEM) proposed by McLeroy et al. (12) and updated by Kumar et al. (13) and McClarty et al. (14). The social ecological model arose in the context of health promotion, in an attempt to understand how health is influenced not only by human behavior but also by environment and social structures (12).

As this program evaluation was done with the primary objective of supporting quality improvement for the WRHA TB program, and included a retrospective analysis of de-identified administrative data, patient consent was not required. The evaluation was conducted in accordance with the Tri Council Policy Statement: Ethical Conduct for Research Involving Humans, Article 2.5, and all interview data were anonymized; therefore no ethics review for the staff interviews was required for this evaluation (15).

## Results and Analysis

### LTBI Screening and Treatment at BridgeCare Clinic

During 2015, Manitoba received 1,767 refugees, with most (97%) moving to Winnipeg. Only a subset of these was classified as government-assisted refugees (694, 39.2%). While the majority of government-assisted refugees receive health care at BridgeCare Clinic, those who are French speaking receive health care at a clinic where Interferon Gamma Release Assay (IGRA) was not available.

BridgeCare Clinic has offered LTBI screening and treatment to refugees since 2014, following the *Canadian Tuberculosis Standards* (2), as part of the primary care provided. Clients have access to basic health screening within 2 weeks of their arrival to Canada. The clinic's primary care nurse (PCN) receives referrals from local settlement agencies. Women and men between 18 and 49 years of age from a TB endemic country, defined as a country experiencing more than 30 cases of TB per 100,000 people every year (2), are eligible for LTBI screening with IGRA as part of their intake. BridgeCare Clinic did not have sufficient resources to screen children or adults outside of this age range for LTBI. From January 2015 to October 2016 1010 refugees were seen at BridgeCare Clinic.

During a follow-up visit with a physician or nurse practitioner 2 weeks later, clients received a complete physical examination and a review of the blood test results. The provider informed the client if the IGRA test was positive, and discussed LTBI care. A chest X-ray was ordered to rule out active TB. Clients with no evidence of active disease were eligible for LTBI treatment; daily self-administered INH and pyridoxine treatment for nine months was offered. Clients were followed monthly, with active follow-up for those who missed appointments. An alanine aminotransferase (ALT) test was performed monthly during the treatment course if the client was older than 35 years, to monitor side effects. If the client was younger than 35 years, the ALT was performed only if hepatotoxicity was suspected.

Education on LTBI is an important part of care at BridgeCare, and takes place at different stages, with written information provided in clients' own languages. Education sessions include information about TB in general, what LTBI is and what it means to have tested positive for LTBI, its treatment, and possible adverse events. The consequences of not treating LTBI were also discussed with clients and their families.

### Screening, Treatment and Outcomes

No clients with active TB are included in our dataset. As part of the LTBI screening program, 274 IGRA tests were ordered between January 2015 and October 2016. IGRA results were negative in 158 clients (57.7%) and positive in 101 (36.9%) (Table 1). The mean age of clients with a negative IGRA test result (29.6 years, SD: 8.8) was significantly lower than the mean age of clients with a positive IGRA test, by 2.9 years (32.5 years, SD: 8.9) (difference 2.9, 95% CI 0.682—5.118, *p* = 0.005). There was no significant difference between proportions of male and female clients with a negative IGRA test result and those with a positive result.

**Table 1:** IGRA test results in adult refugees from January 2015 to October 2016 at BridgeCare Clinic.

**IGRA test result**	***n* = 274, *n* (%)**	**Sex**, ***n*** **(%)**			**Age in years, mean (SD)**
		**Male**	**Female**	**No data**	
Deleted or referred	2 (0.7)	1 (50.0)	1 (50.0)	0	28.0 (1.4)
Canceled	5 (1.8)	1 (20.0)	3 (60.0)	1 (20.0)	34.0 (10.6)
Pending	6 (2.2)	4 (66.7)	2 (33.3)	0	31.5 (11.3)
Unable to be processed	1 (0.4)	0	1 (100.0)	0	22.0
Indeterminate	1 (0.4)	1 (100.0)	0	0	36.0
Negative	158 (57.7)	71 (44.9)	87 (55.1)	0	29.6 (8.8)
Positive	101 (36.9)	45 (44.6)	56 (55.4)	0	32.5 (8.9)
Overall	274 (100.0)	123 (44.9)	150 (54.7)	1 (0.4)	30.8 (9.0)

*IGRA, interferon gamma release assay*.

Clients receiving an IGRA test during the study period were from 23 different countries. Of the 101 clients with IGRA positive results, 89 (88.1%) came from an African country and four countries accounted for 60.2% of clients: the Democratic Republic of the Congo (30.3%), Eritrea (17.5%), Burundi (6.2%), and Liberia (6.2%). Positive IGRA test result proportions in clients from the Eastern Mediterranean, the next largest group of test positives, was 16.5% lower than the proportion from African clients (difference −0.165, CI 95% −0.332—0.003, *p* = 0.038) (Table 2).

**Table 2:** IGRA test results from adult refugees at BridgeCare Clinic, by WHO regions according to the country of birth, January 2015–October 2016.

**WHO regions (country of birth)**	**IGRA test result**	***n* = 274**	**%**
Africa	Negative	121	54.5
	Positive	89	40.1
	Indeterminate	1	0.5
	Unable to be processed	1	0.5
	Pending	6	2.7
	Deleted or referred	2	0.9
	Canceled	2	0.9
	Subtotal	222	81.0
Eastern Mediterranean	Negative	22	73.3
	Positive	7	23.3
	Canceled	1	3.3
	Subtotal	30	10.9
South-East Asia	Negative	13	65.0
	Positive	5	25.0
	Canceled	2	10.0
	Subtotal	20	7.3
Unknown	Negative	2	100.0

*IGRA, interferon gamma release assay; WHO, World Health Organization*.

From January to December 2015, 159 IGRA tests were ordered, and 59 (33%) were positive (Table 3). Of 45 clients eligible for LTBI treatment, 11 (24.4%) declined treatment, and 34 (75.6%) started treatment. Twenty-seven (79.4%) clients completed treatment, 3 (8.8%) clients moved out of province, and 4 (11.8%) did not complete treatment. Of 28 women with IGRA positive results, 12 (42.9%) started treatment; this was significantly lower than the proportion of men that started treatment: 22 out of 31 (71%) (difference −0.281, CI 95% −0.524 to −0.038, *p* = 0.015).

**Table 3:** Sex, WHO region of the country of birth, and LTBI treatment outcome of adult refugees with IGRA test positive January to December 2015 at BridgeCare Clinic.

**Characteristic**		***n* = 59**	**%**	**Mean (SD)**
Age	Age in years, mean (SD)			31.9 (8.8)
Sex	Male	31	52.5	
	Female	28	47.5	
WHO regions (country of birth)	Africa	49	83.1	
	Eastern Mediterranean	6	10.2	
	South-East Asia	4	6.8	
Treatment eligibility	Not a candidate^*^	9	15.3	
	Moved out of province	5	8.5	
	Eligible	45	76.3	
Treatment acceptance (*n* = 45)	Declined treatment	11	24.4	
	Started treatment	34	75.6	
Treatment completion (*n* = 34)	Completed treatment	27	79.4	
	Moved out of the province	3	8.8	
	Did not complete treatment	4	11.8	

*WHO, World Health Organization; LTBI, latent tuberculosis infection; IGRA, interferon gamma release assay; SD, standard deviation*.

* *Reasons included pregnancy, other medical issues, and unspecified*.

**Figure 1:**
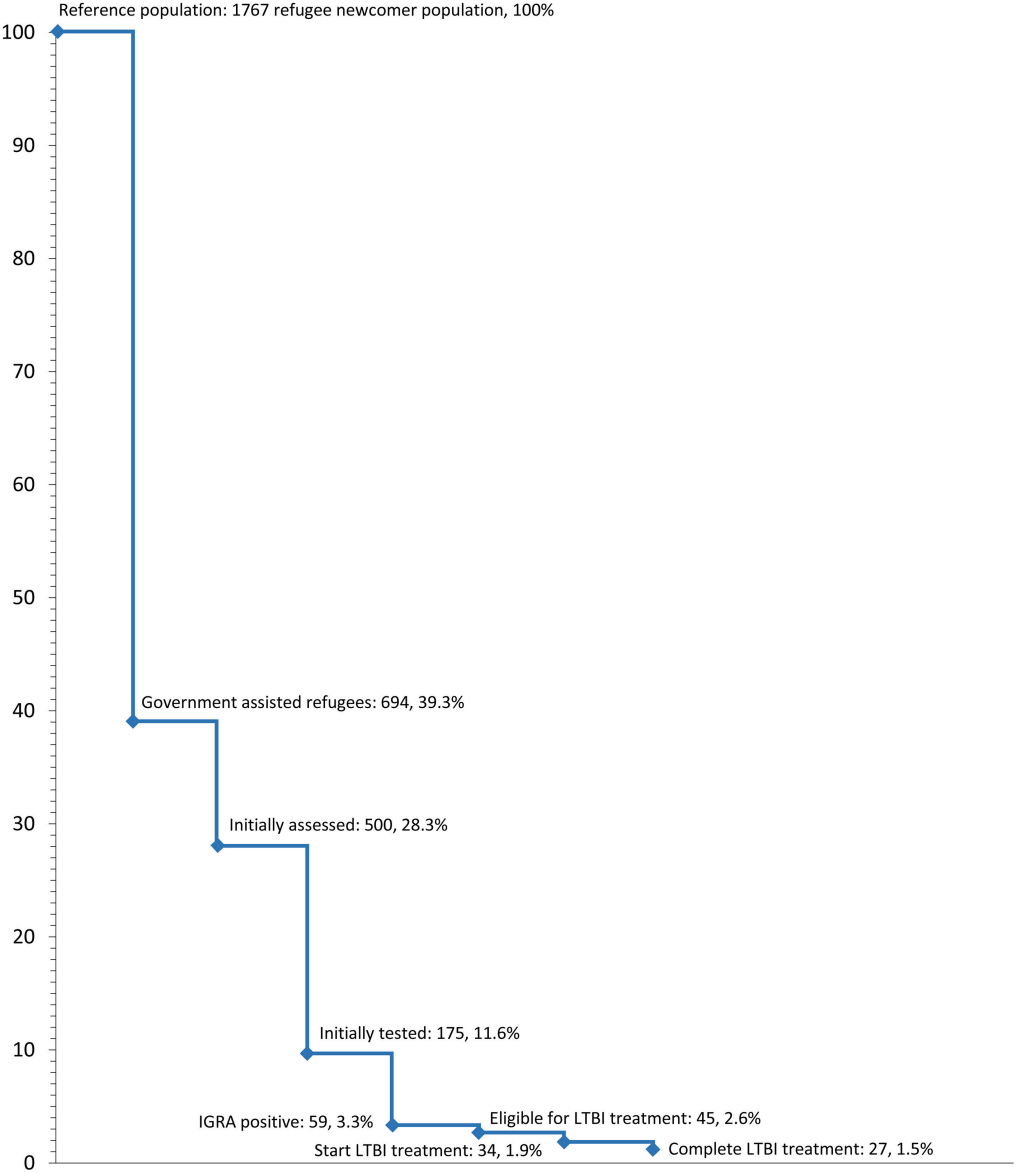
Losses in the cascade of latent tuberculosis infection care in refugee clients at BridgeCare Clinic with positive IGRA and eligible for treatment.

Among all refugees in Manitoba, major losses occur in the group of non-government-assisted (privately sponsored) refugees (Figure 1). Another large loss occurs between those initially assessed and those tested for LTBI. Individuals were not screened after initial assessment because they did not come from a high TB risk country; another large portion of this group includes foreign-born children, most of whom are currently not screened for LTBI in Manitoba.

### Facilitators and Barriers of LTBI Outcomes at BridgeCare Clinic

Interview responses from staff at BridgeCare Clinic were categorized according to the five levels of the social ecological model (Table 4) (12, 14):

1. the *intrapersonal*, characteristics of the individual (e.g., knowledge, behavior, self-concept, etc.);
2. the *interpersonal*, social networks and social support systems (e.g., family, work group, and friendship networks);
3. the *institutional*, includes social institutions, such as health care organizations;
4. the *socio-cultural/community*, relationships among organizations, institutions, and also cultural norms; and
5. the *structural*—policies at all levels

**Table 4:** Facilitators and barriers to successful LTBI treatment completion for 2015–2016, identified in interviews with staff at BridgeCare Clinic.

**Social economic model level (12, 14)**	**Facilitators**	**Barriers**
Intrapersonal	Absence of side effects with first line treatment The low prevalence of alcohol dependence/issues among refugee populations	Younger age of some clients Pregnancy and family planning Unknown age Unknown medical history Language barriers Low literacy levels Concern regarding side effects Long duration of treatment
Interpersonal	Strong relationships with clients	
Institutional	A significant focus on client health education Nurses in central program management roles Clients assigned to a regular primary care physician Accessible and well utilized interpreter service Multipurpose contacts with clients A patient-centered approach to care Improved efficiency and accessibility of laboratory services	Lab services availability Staff and resources limitations Communications across facilities and between providers
Socio-cultural/community	Increased likelihood of refugees having personal experience with active TB patients and fearing disease consequences	Lack of familiarity with prophylactic/preventive medicine
Structural/Policy	The availability and accessibility of IGRA testing Comprehensive health care coverage for refugees (during first year in Canada) Region-wide clinical rounds specific to LTBI	Temporary nature of clinic services Lack of material incentives for treatment completion Limited staff and resources

#### Intrapersonal Level Factors

Two factors at the intrapersonal level were identified by staff as associated with good LTBI treatment. Firstly, few clients reported gastrointestinal intolerance, a common side effect of INH. Adherence to INH is more likely if this relatively mild side effect is not present (16). Secondly, alcohol dependence, which can affect liver toxicity and treatment adherence (17, 18), has been rare among the refugees seen at BridgeCare Clinic (19).

There were a number of intrapersonal level factors noted by staff that hindered LTBI outcomes. In the experience of BridgeCare Clinic staff, *younger clients* find it more difficult to understand the need for medication when they feel well. Often, they do not want to miss classes or alter their school schedules for appointments. Nurses at BridgeCare Clinic try to accommodate their schedules by providing them with appointments late in the day. In some cases clients at BridgeCare Clinic *may not know their own age*, which has implications on their eligibility criteria for LTBI screening.

Other factors which are also age-related include *pregnancy and family planning*. Although INH can be taken during pregnancy (2), there is concern of side effects to the fetus and hepatotoxicity to the woman, mainly during the first three months postpartum (2). For this reason INH treatment is discussed with women during pregnancy at BridgeCare Clinic but LTBI treatment is deferred until 3 months postpartum (2). For those planning to get pregnant, staff discuss LTBI treatment and the woman decides whether to start her treatment or not. Contraception is offered to all women to avoid pregnancy during treatment. If a woman does not wish to use contraception, treatment may be delayed.

Staff commented that it can be very challenging to establish if clients have received active TB treatment in the past, as in some cases the *history is unknown*. A person who received TB treatment in the past may have a positive immune test for *M. tuberculosis* infection, without currently having LTBI. These clients should not be treated for LTBI (2). At BridgeCare, clients are asked about their histories in the first assessment by the primary care nurse, during the physical evaluation by the physician or nurse practitioner, and thirdly during the appointment to start LTBI treatment.

*Concern about side effects* can be another barrier for treatment acceptance. Although there are some side effects related to LTBI treatment, staff commented that the concern among clients is often disproportionate to the documented risk. The nine month course of INH is perceived to be a *long treatment period* for some clients and is a barrier to treatment acceptance and completion. During the evaluation period there were discussions at BridgeCare Clinic about the possibility of offering alternative shorter-course treatments (e.g., four months of rifampin).

Even with the interpreter service, *language* still can be an issue. For some languages it is very difficult to find an interpreter, which can affect communication with the client. There is also a wide spectrum of *literacy* among BridgeCare Clinic clients. When health literacy is low, it is more difficult for staff to explain important health concepts such as the meaning of LTBI, and the difference between LTBI and active disease.

#### Interpersonal Level Factors

At an interpersonal level, the *relationships* established between health care providers and clients over many visits for various purposes, including physical examinations and vaccine catch up were seen by staff as a strong facilitator for good treatment outcomes. Our analysis found no interpersonal level barriers to treatment from interview data.

#### Institutional Level Factors

The integrated care provided at BridgeCare was considered by staff to have facilitated LTBI treatments in a number of ways at the institutional level. Clients receive *health education* from both physicians and nurses, and written information is also provided, so that information is consistently reinforced. Health education can help increase treatment adherence. Additionally, BridgeCare Clinic had *interpreter services* available in many languages for clients, facilitating communication.

The program is strongly influenced by *nursing care models* which tend to emphasize a holistic approach to disease, and focus on the changing needs and situation of each individual patient. At the same time, clients are assigned a *regular primary care physician*, involved in other screening and health services. According to the staff interviewed, this facilitates better LTBI treatment completion outcomes than systems where clients receive partial and ephemeral care from multiple health care providers (20–22). Having *multi-purpose contacts* with clients provides the opportunity to enhance LTBI education, alleviate concerns, and improve treatment adherence.

On-site *laboratory sampling* was opened in January 2016 in BridgeCare Clinic. Integration of the lab enables a “One Stop Shop” approach, and was seen by staff as leading to fewer canceled or rescheduled IGRA tests. Despite improvements in laboratory services accessibility, there are still limits to the days and times when blood sampling for IGRA tests can occur. This is primarily due to the availability of the referral lab and technical issues inherent to the test.

In all, the staff at BridgeCare Clinic actively follow a *patient-centered approach* to care. They try to establish good relationships with clients by taking into account their personal needs. This includes access to interpreter services, the provision of health education, and the provision of primary health care to clients' families, which stimulates family involvement.

As a comprehensive refugee clinic, *resource limitations* are always an issue. The availability of staff to respond in a timely manner to patient needs is related to the number of government-assisted refugees regularly arriving in Winnipeg. Initially, the number of staff at BridgeCare Clinic was sufficient to serve approximately 500 people each year, without screening children for LTBI. In 2016, the budget was increased to serve approximately 650 clients per year, but the greater number of refugees arriving to the province means staff time and resources are still stretched. Resource limitations have also prohibited the inclusion of services such as screening for children as mentioned and dedicated LTBI program follow-up calls.

Additionally, *communication across facilities and between providers creates challenges*. Pre-immigration chest X-ray results are not available for BridgeCare Clinic staff. In the case that a person has an abnormal pre-immigration chest X-ray (except those consistent with active TB) a respirologist at a tertiary care hospital receives a letter from the Public Health Agency of Canada, and a similar letter is given to the assessed person. Communication gaps or errors can occur when another provider or the person with the abnormal chest X-ray result do not inform BridgeCare Clinic staff of this development. This may result in a duplication of services.

#### Socio-Cultural/Community Level Factors

At the socio-cultural/community level, staff commented on the likelihood that refugees *know people with active TB*, and have witnessed some of the suffering caused by the disease, including death. According to the staff interviewed, these experiences contribute to clients' fear of the disease and their willingness to start and complete treatment. On the other hand, the concept of taking medication for the purpose of *prevention* is not a familiar one among many refugee clients at BridgeCare Clinic, and was seen as a barrier at the this level. This unfamiliarity with prophylactic or preventive medication can affect LTBI acceptance rates, as clients may not understand why they should take medication when they do not feel sick.

#### Structural/Policy Level Factors

BridgeCare Clinic staff noted three factors at the Structural/Policy level that facilitated LTBI treatment. Government-assisted refugees can receive *comprehensive health care* at BridgeCare Clinic during their first year in Canada at no cost. This includes basic screening, outreach worker supports, and vaccine catch up, among others. The availability and *accessibility of IGRA testing* is provided in-kind by Cadham Provincial Laboratory. According to the literature, IGRA performs better than TST among certain populations like refugees (23). Advantages to IGRA include: the need for only one visit for blood sampling, with a significantly wider period of time for sampling during the week (rather than two visits for a TST application and reading, with limited time and days for sampling); IGRA testing has higher specificity, especially in individuals previously vaccinated with BCG (increased likelihood in refugee populations); IGRA test interpretation is systematic and does not involve as many subjective steps as TST. Staff at BridgeCare Clinic found IGRA to be more convenient, effective, and easier to interpret than TST. Thirdly, the WRHA hosts *monthly rounds on integrated LTBI care* to support clinician decision making, communication and application of guidelines. They also present opportunities for discussions on improving the client care system. BridgeCare Clinic staff regularly attend these meetings and have the opportunity to discuss challenging LTBI cases with colleagues and experts, as well as to be updated on emerging research and practices

Health care for refugees at BridgeCare Clinic ends after the first year of arrival to Canada. At that time, clients are referred to a different primary care clinic for their health care needs. This *temporary care* presents some challenges to clients trying to complete the LTBI screening and treatment before they move to another clinic and potentially compromise their continuity of care (especially with a nine month INH regimen). Additionally, staff cited a *lack of incentives* as hindering care completion. Evidence indicates that the provision of incentives to individuals requiring treatment for active TB or LTBI, such as foods, money, celebrations, etc., enhances treatment acceptance and completion rates (24–26). There are not sufficient resources to support this kind of strategy at BridgeCare Clinic. *Staff and resource limitations* can be a challenge at the structural/policy level in addition to the institutional level.

## Discussion

Our evaluation found a treatment acceptance rate of ~ 76% and completion rate between 79 and 88% (upper estimate based on successful completion by those out of province). These estimates compare favorably to those found by Manitoba Health, Seniors, and Active Living, indicating promising treatment completion outcomes at primary care sites for non-complex LTBI care (including BridgeCare Clinic) (7). In comparison, a systematic review published in 2008 on adherence to LTBI treatment in the US and Canada describes completion rates among the foreign-born populations ranging from 22% (in a study of pregnant women) to 90%, depending on study size and population (5).

The LTBI program at BridgeCare Clinic is an integrated program managed mainly by primary care practitioners, part of their patient-centered care informed by nursing models. Integrating LTBI care with primary care can facilitate multipurpose contacts with a client and help develop strong relationships between the client and health care provider. Staff from the clinic actively reach out to clients instead of waiting for clients to contact them. The staff feel that the interpretation service contributes to a more culturally safe approach and improves communication between providers and clients. Client education also plays a very important role. Finally, the availability of laboratory sampling at the clinic, free IGRA testing, and efficient lab systems have contributed to treatment success.

The most recent WHO strategy for TB control, The End TB Strategy, calls for integrated, patient-centered care and prevention in TB programming (4). WHO suggests that National Tuberculosis Programs incorporate social supports into clinical care and that treatment should include social protection measures, nutritional support, and decentralized TB care. These recommendations align with the WRHA's move to more decentralized care for TB, and the patient-centered model at BridgeCare Clinic which provides interpreter services, and integrated medical care for individuals and families.

There were some barriers to successful LTBI treatment and care for refugees at BridgeCare Clinic, many of which are identified in the *Guidance for Tuberculosis Prevention and Control Programs in Canada* (3). These include linguistic, cultural, socio-economic and health service gaps. While resource limitations can be seen as a challenge in any health program, recent increases in refugee arrivals to Manitoba have made dedicated staff time for LTBI management and lab accessibility for LTBI screening scarcer.

Other studies on treatment outcomes seen in comparable TB care settings and systems produced mixed findings. While many studies produced evidence in support of program decentralization and the integration of TB management with primary care for priority populations (27–30), a number of other investigations found poorer treatment outcomes from integrated models of care (31–33). Authors attributed poor outcomes to factors that may have been specific to the particular centers being studied (e.g., personnel issues), but further research is required to assess what types of conditions facilitate success and what conditions produce challenges when integrating TB management with primary care. This study begins to address these questions.

LTBI treatment aims to reduce the risk of progressing to active TB, however according to previous studies the estimated number needed to treat to prevent one case of LTBI from progressing to active tuberculosis would range from 111 to 314 (depending on the patient's risk for progression) (34), suggesting that the number that complete therapy through the program provided at BridgeCare Clinic may have limited impact on its own.

The treatment cascade illustrates that there is no information available regarding LTBI screening and treatment for ~ 60% of refugees arriving in Manitoba. It is also widely recognized that children, especially those under 5 years of age, have a higher risk of progressing from infection to active TB. However, BridgeCare Clinic does not have capacity to screen children for LTBI, nor non-government sponsored refugees. Even with good treatment acceptance and completion rates (around 80%), there is still a large proportion of losses for treatment initiation. These losses include moving from the province, fear of side effects especially by pregnant women and women planning to be pregnant, among others. Another proportion of losses occurs because treatment is not completed. Movement from the province, and factors such as the presence of side effects contribute to these losses; mis-conceptions about LTBI treatment may also explain some of the losses to the treatment cascade.

Descriptions of facilitators and barriers of LTBI outcomes at BridgeCare Clinic were gathered during the interviews with staff. Our study is limited by not having evidence from client interviews about their own perceptions of what enables or hinders their LTBI treatment.

Improving availability of free LTBI screening using IGRA for all refugees (not only government-assisted refugees), including children, will help to achieve improved population level outcomes among those with LTBI and will also improve reductions of the LTBI reservoir in foreign born persons in Manitoba. Although the findings of this study support the success of decentralizing TB services, it is critical that ongoing monitoring and evaluation continues to ensure that success of LTBI treatment completion is maintained over time. Since this program evaluation, BridgeCare Clinic has switched to almost exclusive use of rifampin over four months or rifapentine/isoniazid once weekly over three months, a development that warrants further program evaluation.

A decentralized/primary care integrated approach can be effective, as demonstrated here. Aligned with WHO recommendations, our experience suggests that LTBI care and treatment can be delivered effectively in the primary care setting using an integrated, patient-centered approach.

## Data Availability

The datasets for this manuscript are not publicly available because data were from BridgeCare Clinic in Winnipeg and are not part of any public datasets. Requests to access the datasets should be directed to pplourde@wrha.mb.ca.

## Author Contributions

DB-B: conceptualization, methodology, formal analysis, investigation, data collection and writing-original draft, review, and editing. MB and YK: conceptualization, methodology, resources, writing-review and editing, and supervision. MH-B: conceptualization, resources, writing-review and editing, and project administration. SB: conceptualization, methodology, investigation, writing-review and editing. KH: investigation, resources, data collection and curation, writing-review and editing. J-AL: resources, data collection and curation, writing-review and editing. AB and PP: conceptualization, resources, and writing-review and editing.

### Conflict of Interest Statement

The authors declare that the research was conducted in the absence of any commercial or financial relationships that could be construed as a potential conflict of interest.

## References

[1] Vachon J Gallant V Siu W Tuberculosis in Canada,2016. Can Commun Dis Rep. (2018) 44:75–81. 10.14745/ccdr.v44i34a01 PMC6449093 31007614

[2] Public Health Agency of Canada, The Lung Association Canadian Thoracic Society. Canadian Tuberculosis Standards, 7th edn. Ottawa: Public Health Agency of Canada (2014). 465 p

[3] Pan-Canadian Public Health Network Guidance for Tuberculosis Prevention and Control Programs in Canada - Pan-Canadian Public Health Network. Ottawa (2012). Available online at: http://www.phn-rsp.ca/pubs/gtbpcp-oppctbc/pdf/Guidance-for-Tuberculosis-Prevention-eng.pdf (Accessed Oct 11, 2018).

[4] World Health Organization Implementing the End TB Strategy: The Essentials. Geneva: World Health Organization (2015). 113 p. Available online at: http://www.who.int/tb/publications/2015/end_tb_essential.pdfua=1

[5] Hirsch-Moverman Y Daftary A Franks J Colson PW. Adherence to treatment for latent tuberculosis infection: systematic review of studies in the US and Canada. Int J Tuberc Lung Dis. (2008) 12:1235–54. 18926033

[6] Winnipeg Regional Health Authority Integrated Tuberculosis Services. Strategic Planning Workbook. (2012). 48 p. Available online at: http://www.wrha.mb.ca/prog/tuberculosis/files/IntegratedTBWorkbookFinalJan2012.pdf

[7] Manitoba Health Seniors and Active Living Distribution and Completion of Treated Latent Tuberculosis Infection in Winnipeg. (2016). 26 p. Available online at: https://www.gov.mb.ca/health/publichealth/surveillance/docs/ltbi.pdf

[8] Manitoba Centre for Health Policy University of Manitoba - Development &amp; Advancement - Term: Drug Program Information Network (DPIN). (2018). Available onlien at: http://mchp-appserv.cpe.umanitoba.ca/viewDefinition.php.definitionID=102596. (Accessed Nov 7, 2018)

[9] Creswell JW Research design. In: Creswell JW Creswell JD, editors. Qualitative, quantitative, and mixed methods approaches. 5th ed. Thousand Oaks, CA: SAGE Publications Inc (2018). p. 275.

[10] World Health Organization Global Tuberculosis Report 2016. Geneva: World Health Organization (2016). 201 p. Available online at: http://apps.who.int/medicinedocs/es/m/abstract/Js23098en/

[11] Alsdurf H Hill PC Matteelli A Getahun H Menzies D. The cascade of care in diagnosis and treatment of latent tuberculosis infection: a systematic review and meta-analysis. Lancet Infect Dis.(2016) 16:1269–78. 10.1016/S1473-3099(16)30216-X 27522233

[12] McLeroy KR Bibeau D Steckler A Glanz K. An ecological perspective on health promotion programs. Health Educ Q. (1988) 15:351–77. 10.1177/109019818801500401 3068205

[13] Kumar S Quinn SC Kim KH Musa D Hilyard KM Freimuth VS. The social ecological model as a framework for determinants of 2009 H1N1 influenza vaccine uptake in the United States. Heal Educ Behav. (2012) 39:229–43. 10.1177/1090198111415105 21984692 PMC3916095

[14] McClarty LM Lorway RR Ramanaik S Wylie J Becker ML Factors influencing frontline health service providers' likelihood to recommend a future, preventive HIV vaccine to key populations in Karnataka, south India. Vaccine. (2015) 29 33:656–63. 10.1016/j.vaccine.2014.12.009 25528520

[15] Government of Canada IAP on RE Interagency Advisory Panel on Research Ethics. Available online at: http://www.pre.ethics.gc.ca/eng/policy-politique/initiatives/tcps2-eptc2/chapter2-chapitre2/#toc02-1a (Accessed Nov 7, 2018)

[16] Denholm JT McBryde ES Eisen DP Penington JS Chen C Street AC. Adverse effects of isoniazid preventative therapy for latent tuberculosis infection: a prospective cohort study. Drug Healthc Patient Saf. (2014) 6:145–9. 10.2147/DHPS.S68837 25364275 PMC4211866

[17] Brudney K Dobkin J. Resurgent tuberculosis in New York City: Human Immunodeficiency Virus, homelessness, and the decline of tuberculosis control programs. Am Rev Respir Dis. (1991) 144:745–9. 10.1164/ajrccm/144.4.745 1928942

[18] Pablos-Méndez A Knirsch CA Barr RG Lerner BH Frieden TR. Nonadherence in tuberculosis treatment: predictors and consequences in New York City. Am J Med. (1997) 102:164–70. 10.1016/S0002-9343(96)00402-0 9217566

[19] Beiser M Health of immigrants and refugees in Canada, the (Commentary). Can J Public Heal. (2005) 96:30–44. 10.1007/BF03403701 PMC6976041 16078554

[20] World Health Organization Adherence to Long-Term Therapies: Evidence for Action. Geneva: World Health Organization (2003). Available online at: http://www.who.int/chp/knowledge/publications/adherence_report/en/. (Accessed Nov 8, 2018)

[21] Simone PM Essential components of a tuberculosis prevention and control program: recommendations of the advisory council for the elimination of tuberculosis. Morb Mortal Wkly Rep Recomm Rep. (1995) 44:1–16. 7565539

[22] Centers for Disease Control and Prevention Managing Tuberculosis Patients and Improving Adherence. Atlanta. (2014). Available online at: https://www.cdc.gov/tb/education/ssmodules/pdfs/Module6v2.pdf

[23] Campbell JR Krot J Elwood K Cook V Marra F. A systematic review on TST and IGRA tests used for diagnosis of LTBI in immigrants. Mol Diagn Ther. (2015) 19:9–24. 10.1007/s40291-014-0125-0 25579159

[24] Lutge EE Wiysonge CS Knight SE Volmink J. Material incentives and enablers in the management of tuberculosis. Cochrane Database Syst Rev. (2012) 1:CD007952. 10.1002/14651858.CD007952.pub2 22258976

[25] Beith A Eichler R Weil D Worldwide: Incentives for tuberculosis diagnosis and treatment. In: Eichler R Levine R the Performance-Based Incentives Working Group, editors. Performance Incentives for Global Health: Potential and Pitfalls. Washington, DC: Center for Global Development (2009). p. 237–56.

[26] Latent tuberculosis infection: a guide for primary health care providers Centers for Diseases Control and Prevention. Atlanta, GA (2013).

[27] Rennert-May E Hansen E Zadeh T Krinke V Houston S Cooper R. A step toward tuberculosis elimination in a low-incidence country: successful diagnosis and treatment of latent tuberculosis infection in a refugee clinic. Can Respir J. (2016) 2016:7980869. 10.1155/2016/7980869 27445565 PMC4904499

[28] Subedi P Drezner KA Dogbey MC Newbern EC Yun K Scott KC. Evaluation of latent tuberculous infection and treatment completion for refugees in Philadelphia, PA, 2010-2012. Int J Tuberc Lung Dis. (2015) 19:565–9. 10.5588/ijtld.14.0729 25868025

[29] Tavitian SM Spalek VH Bailey RP. A pharmacist-managed clinic for treatment of latent tuberculosis infection in health care workers. Am J Health Syst Pharm. (2003) 60:1856–61. 10.1093/ajhp/60.18.1856 14521037

[30] el-Sadr W Medard F Berthaud V. Directly observed therapy for tuberculosis: the Harlem Hospital experience, 1993. Am J Public Health. (1996) 86:1146–9. 10.2105/AJPH.86.8_Pt_1.1146 8712276 PMC1380628

[31] Rubinowicz A Bartlett G MacGibbon B Greenaway C Ronald L Munoz M. Evaluating the role of primary care physicians in the treatment of latent tuberculosis: a population study. Int J Tuberc Lung Dis. (2014) 18:1449–54. 10.5588/ijtld.14.0166 25517810

[32] Ignotti E Oliveira de BFA Hartwig S Oliveira de HC Scatena JHG. Analysis of the Tuberculosis Control Program in the city of Caceres, Brazil, prior to and after the implementation of a family health program. J Bras Pneumol. (2007) 33:287–94. 10.1590/S1806-37132007000300010 17906790

[33] Chung W-S Chang R-E Guo H-R. Variations of care quality for infectious pulmonary tuberculosis in Taiwan: a population based cohort study. BMC Public Health. (2007) 7:107. 10.1186/1471-2458-7-107 17562022 PMC1906756

[34] Force USPST Bibbins-Domingo K Grossman DC Curry SJ Bauman L Davidson KW Screening for latent tuberculosis infection in adults: us preventive services task force recommendation statement. JAMA. (2016) 316:962–9. 10.1001/jama.2016.11046 27599331

